# Primary ovarian leiomyoma associated with endometriotic cyst presenting with symptoms of acute appendicitis: a case report

**DOI:** 10.1186/1746-1596-4-25

**Published:** 2009-07-30

**Authors:** Davor Tomas, Tanja Leniček, Neven Tučkar, Zvonimir Puljiz, Mario Ledinsky, Božo Krušlin

**Affiliations:** 1 Department of Pathology, Sestre Milosrdnice University Hospital, Zagreb, Croatia; 2 Department of Gynecology and Obstetrics, Sestre Milosrdnice University Hospital, Zagreb, Croatia; 3 Department of Surgery, Sestre Milosrdnice University Hospital, Zagreb, Croatia; 4 University of Zagreb, School of Medicine, Zagreb, Croatia

## Abstract

**Background:**

Ovarian leiomyoma is a rare benign tumor that accounts for 0.5 to 1% of all benign ovarian tumors. It probably arises from smooth muscle cells in the ovarian hilar blood vessels but there are other possible origins including cells in the ovarian ligament, smooth muscle cells or multipotential cells in the ovarian stroma, undifferentiated germ cells, or cortical smooth muscle metaplasia. Additionally, smooth muscle metaplasia of endometriotic stroma, smooth muscle present in mature cystic teratomas, and smooth muscle in the walls of mucinous cystic tumor may explain their occurrence in the ovary in some cases.

**Case presentation:**

A 31-year-old woman was admitted to our surgical emergency service with a one-day history of appendicitis-like symptoms. Upon laparotomy, there was a solid, oval left-sided ovarian tumor located behind the uterus. The tumor was sent to the pathology department. A diagnosis of primary ovarian leiomyoma associated with an endometriotic cyst was established.

**Conclusion:**

The origin of ovarian leiomyoma is still unresolved. In our case, the tumor probably arose from smooth muscle cells derived from myofibroblasts that originate from metaplastic ovarian stromal cells present in the rim of the endometriotic cyst. Despite its rarity, ovarian leiomyoma should be considered in the differential diagnosis of ovarian spindle cell tumors. Appropriate diagnosis may require additional immunohistochemical analysis in some cases.

## Background

Ovarian leiomyoma is a rare benign tumor that accounts for 0.5 to 1% of all benign ovarian tumors [1]. Most of these tumors are unilateral, measure only few millimeters in diameter and generally occur in premenopausal women [2]. However, in the pediatric/young adult group they are more commonly bilateral and no bilateral cases have been described in patients over the age of 35 [3].

They probably arise from smooth muscle cells in the ovarian hilar blood vessels but other possible origins include cells in the ovarian ligament, smooth muscle cells or multipotential cells in the ovarian stroma, undifferentiated germ cells, or cortical smooth muscle metaplasia [4-6]. Additionally, smooth muscle metaplasia of endometriotic stroma, smooth muscle present in mature cystic teratomas, and smooth muscle in the walls of mucinous cystic tumor may explain their occurrence in the ovary in some cases, as suggested by some of the association seen in the current case and in other reports [7-11].

We report a rare case of a primary ovarian leiomyoma associated with an endometriotic cyst that caused acute abdomen, mimicking acute appendicitis.

## Case presentation

A 31-year-old woman was admitted to our surgical emergency service with a one-day history of intermittent abdominal pain associated with fever, nausea and vomiting. Her history was unremarkable. On physical examination, she was found to have right lower quadrant abdominal tenderness with guarding. The white blood cell count was 12800/mm^3^, and C-reactive protein was elevated. An ultrasound examination on the abdomen showed free fluid in right ileocecal region. Acute appendicitis was suspected and the patient underwent an urgent surgical procedure. Upon laparotomy, free brownish fluid in the peritoneum and a hyperemic appendix were detected. Inspection of the uterus and ovary revealed a solid, oval left-sided ovarian tumor located behind the uterus. The tumor was distinctly separated from the uterus. There was no adhesion or infiltration of the surrounding structures. The right salpinx, ovary and uterus were normal by inspection and were left intact. Left sided salpingo-oophorectomy and appendectomy was performed.

**Figure 1:**
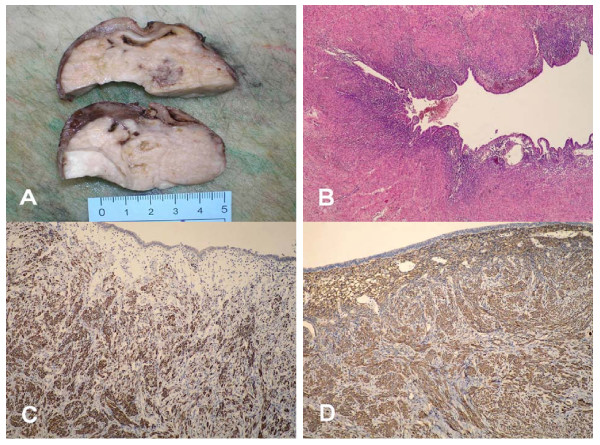
**Grossly, the tumor was white-gray in color and the cut surface displayed a whorled pattern**. On the pole beneath the salpinx there was a cyst measuring 3 cm in greatest diameter filled with brownish material that was included in the tumor (A). Microscopically, the tumor was composed of whorled interlacing fascicles of typical smooth muscle cells, and the lumen of the cyst was covered with cuboidal epithelial cells surrounded by endometrial-type stroma (B, 40×, H&E). Immunohistochemically, the tumor cells were positive for desmin (C, 100×) and α-smooth muscle actin (D, 100×).

The tumor was firm, had a smooth and shiny surface and measured 11 × 10 × 6 cm. The cut surface of the tumor was white-gray in color and displayed a whorled pattern. On the pole beneath the salpinx there was a cyst measuring 3 cm in greatest diameter filled with brownish material that was included in the tumor (Fig. 1A). In the vicinity of the cyst, there were signs of hemorrhage and a thin, brownish, coarse layer of fibrin covered the tumor surface in this area. Grossly, there was no recognizable normal ovarian tissue. The left salpinx was unremarkable. The serosal surface of the appendix, which measured 4.5 × 1 cm, was opacified.

Microscopically, the tumor was composed of whorled interlacing fascicles of typical smooth muscle cells with bland nuclei. Focally, there was dense fibrosis and hyalinization but significant nuclear atypia, pleomorphism and necrosis were absent. Mitotic activity was sparse (up to 1/10 high power field). The lumen of the cyst was covered with cuboidal epithelial cells without signs of atypia, surrounded by endometrial-type stroma (Fig. 1B). There were siderophages and extravasated red blood cells in the vicinity of the cyst. Above the cyst, there were fibrin, red blood cells and polymorphonuclears on tumor surface. Multiple sections showed no additional stromal or glandular elements in the tumor. The serosal surface of the appendix and surrounding fat tissue were infiltrated with polymorphonuclears, but there were no signs of appendicitis.

Immunohistochemically, the tumor cells were positive for vimentin, desmin (Fig. 1C) and α-smooth muscle actin (Fig. 1D) but not for CD34, S100 protein, epithelial membrane antigen, α-inhibin, calretinin and CD117. Epithelial lining of the cyst was positive for epithelial membrane antigen and the surrounding stroma was positive for CD10 (all antibodies from DAKO; Copenhagen, Denmark).

The diagnosis of primary ovarian leiomyoma associated with endometriotic cyst and periappendicitis was established.

The patient had no complication during the postoperative course and was discharged 7 days after surgery. No further therapy was indicated, and patient is well, 6 months after the diagnosis was made.

## Discussion

Ovarian leiomyoma is frequently associated with other ipsilateral or contralateral ovarian lesions [7-10] but to our knowledge this is the first case of a primary ovarian leiomyoma associated with an endometriotic cyst.

A similar case of ovarian adenomyoma associated with endometriotic cyst was described by McDougal and Roth [11]. An adenomyoma presenting outside the uterus is an extremely rare entity. Adenomyoma is a circumscribed tumorlike mass consisting of endometroid glands, stroma, and smooth muscle tissue [11,12]. In our case multiple sections showed no any additional stromal or glandular element in tumor and diagnosis of adenomyoma was excluded.

Possible origin of this leiomyoma and adenomyoma may be smooth muscle metaplasia in an ovarian endometriotic cyst. Fukunaga [7] showed that smooth muscle metaplasia in ovarian endometriosis is not a rare event and that its frequency was about 18%. Two basic types of smooth muscle metaplasia in ovarian endometriosis were described. In the first type, short fascicles or stellate foci of metaplastic smooth muscle were present in the stroma of endometriosis. In the second type, the endometriotic cyst was surrounded with an incomplete rim of smooth muscle cells [7].

There are two proposed theories trying to explain the presence of smooth muscle in ovarian endometriosis. First, smooth muscle could originate from metaplastic endometrial stromal cells in endometriotic foci [7]. Second, smooth muscle cells could be derived from myofibroblasts that originate from metaplastic ovarian stromal cells present in the rim of the endometriotic cyst [13]. In our case, the second theory sounds more plausible because we did not find foci of smooth muscle metaplasia in endometrial stroma nor smooth muscle cells of leiomyoma completely surrounding the endometriotic cyst.

Despite a relatively high incidence of smooth muscle metaplasia in ovarian endometriosis, so far there have been no reports of leiomyoma clearly arising from endometriosis.

Most ovarian leiomyomas are asymptomatic and are found either during routine physical examination, incidentally at surgery, or at autopsy [1-5,10]. In symptomatic cases, a variety of clinical presentations have been described, such as: abdominal pain, a palpable mass, hydronephrosis, elevated CA-125, hydrothorax and ascites [10,14-16].

In our case, symptoms were most likely caused by the endometriotic cyst present in leiomyoma, than the leiomyoma itself. Localization of the tumor behind the uterus compromised visualization of the tumor by ultrasound and caused appendicitis-like symptoms.

The features of ovarian leiomyoma are very characteristic, but due to its rarity several other tumors were included in the differential diagnosis. The main differential diagnostic considerations for ovarian leiomyoma include sex-cord stromal tumors, such as fibroma/thecoma, particularly if there is a large amount of stromal fibrosis or if the tumors are small. Finally, leiomyosarcoma obviously has to be excluded in this setting. [1,17,18].

Immunohistochemical analysis to demonstrate smooth muscle differentiation, especially desmin, can usually be helpful in distinction between leiomyomas and fibromatous tumors. Desmin shows diffuse positivity in leiomyomas, whereas fibromatous tumors are typically negative or only focally positive. Smooth muscle actin is often positive in both leiomyomas and fibromatous tumors and it is not useful in differential diagnosis [19]. Cellular thecoma could be also considered in differential diagnosis but thecoma does not express smooth muscle actin and expresses α-inhibin and calretinin [20].

Ovarian leiomyomas must be also differentiated from leiomyosarcomas but due to the rarity of these tumors histologic features of malignancy have not been well defined. Pathologists have traditionally used criteria that stress the mitotic count, but it is evident that some other criteria, such as cytological atypia and tumor necrosis must be used when considering the possibility of malignancy in a smooth muscle uterine tumor [17,18].

## Conclusion

Ovarian leiomyoma is a very rare tumor of unresolved origin. In our case, the tumor probably arose from smooth muscle cells derived from myofibroblasts that originate from the ovarian stromal cells present in the rim of the endometriotic cyst. Despite its rarity, ovarian leiomyoma should be considered in the differential diagnosis of ovarian spindle cell tumors. Appropriate diagnosis in some cases requires extensive tumor sampling and additional immunohistochemical analysis.

## Consent

Written informed consent was obtained from the patient for publication of this case report and accompanying images. A copy of the written consent is available for review by the Editor-in-Chief of this journal.

## Competing interests

The authors declare that they have no competing interests.

## Authors' contributions

DT participated in the histopathological evaluation, acquired photomicrographs and drafted the manuscript. TL participated in the histopathological evaluation and drafted the manuscript. ZP and ML were involved in literature search and preparing the material. NT suplyed relevant clinical information about the patient and was involved in manuscript revision. BK outlined the general concept of the manuscript, has been involved in drafting and revising it critically. All authors have read and approved the final manuscript.
